# Discovery of *Pantoea rodasii* Strain ND03 that Produces *N*-(3-Oxo-hexanoyl)-l-homoserine Lactone

**DOI:** 10.3390/s140509145

**Published:** 2014-05-23

**Authors:** Nina Yusrina Muhamad Yunos, Wen-Si Tan, Nur Izzati Mohamad, Pui-Wan Tan, Tan-Guan-Sheng Adrian, Wai-Fong Yin, Kok-Gan Chan

**Affiliations:** Division of Genetics and Molecular Biology, Institute of Biological Sciences, Faculty of Science, University of Malaya, 50603 Kuala Lumpur, Malaysia; E-Mails: ninayusrina@hotmail.com (N.Y.M.Y.); tmarilyn36@gmail.com (W.-S.T.); zetty_mohamad@yahoo.com (N.I.M.); acelinetan38@yahoo.com (P.-W.T.); adrian_tan_1991@yahoo.com (T.-G.-S.A.); yinwaifong@yahoo.com (W.-F.Y.)

**Keywords:** cell-to-cell communication, mass spectrometry, triple quadrupole liquid chromatography mass spectrometry, quorum sensing, *N*-(3-oxohexanoyl) homoserine-lactone (3-oxo-C6-HSL), *Pantoea rodasii*

## Abstract

Proteobacteria use quorum sensing to regulate target gene expression in response to population density. Quorum sensing (QS) is achieved via so-called signalling molecules and the best-studied QS signalling system uses *N*-acyl homoserine lactones (AHLs). This study aimed to identify and characterize the production of AHLs by a bacterium ND03 isolated from a Malaysian tropical rainforest waterfall. Molecular identification showed that ND03 is a *Pantoea* sp. closely related to *Pantoea rodasii*. We used *Chromobacterium violaceum* CV026, an AHL biosensor for preliminary AHL production screening and then used high resolution triple quadrupole liquid chromatography-mass spectrometry, to confirm that *P. rodasii* strain ND03 produced *N*-(3-oxo-hexanoyl)-l-homoserine lactone (3-oxo-C6-HSL). To the best of our knowledge, this is the first report for such a discovery in *P. rodasii* strain ND03.

## 1. Introduction

Many bacteria in association with plants or animals utilize cell-to-cell communication to monitor their virulence determinants [1]. The discovery of bacteria cell-to-cell communication (quorum sensing (QS)) started at the late 1960s, when *Vibrio fischeri* was the first organism discovered to coordinate luminescence by cell-to-cell communication [2]. QS was later confirmed as a bacteria communication system in response towards population density whereby when the threshold level of a signaling molecule is obtained [3,4], then a battery of QS-mediated genes will be regulated by QS [5].

**Figure 1.**
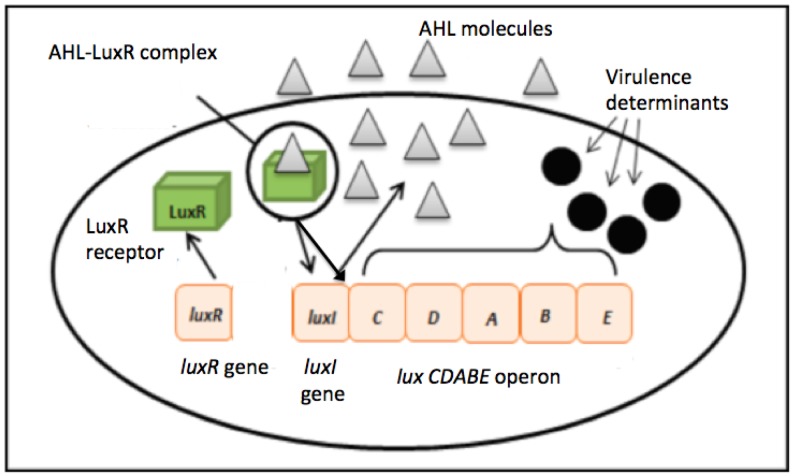
Autoinducer type 1 quorum sensing signal system in Gram-negative bacteria that employ LuxI/LuxR complex for communication.

Two major QS system signaling molecules namely autoinducer-1 *N*-acyl homoserine lactone (AHL)-based system commonly used by most Gram-negative bacteria and autoinducer-2, oligopeptide-based system used by most Gram-positive bacteria [6,7]. The formal has been widely studied where LuxR is a transcriptional activator protein that binds to specific AHL produces by LuxI, the autoinducer synthase (Figure 1) [6,8]. When a critical threshold of population density concentration is achieved, the signal transduction cascade will be activated and allow the target genes to be modulated and a collective behavior adaptation will occur [9,10].

To date, there are different pathogens that possess QS activity where they could be either animal or plant-pathogens. The species clustered in the genus *Pantoea* have been documented to show QS properties, but their possible association with hosts and disease remain unclear, for example, the role of QS in *Pantoea stewartii* that infects corn [11]. Another example is *Pantoea agglomerans*, which is also known to possess QS properties and causes infections in wounds, blood and the urinary tract [12]. The pathogenic effect is believed to be influenced by regulation of virulence determinants throughout the infection process and QS allows these pathogenic bacteria to make a concerted attack and produce ample virulence factors to overwhelm the host defenses [13]. Hence, this triggered interest in our group to extend this research in isolating environmental water-borne bacteria that possess QS properties. In this study, we investigated the presence of QS bacteria in a Malaysian tropical rainforest waterfall sample, and report the isolation of a QS bacterium, *Pantoea rodasii* ND03.

## 2. Experimental Section

### 2.1. Collection of Water Samples

The sampling location was performed at Sungai Ampang waterfalls, Ulu Klang Selangor, Malaysia (GPS coordinate: N03 12.69′ E101 47.72′) and the samples were collected in October 2013. The samples were collected at a depth of 12–17 cm and stored in sterilised plastic tubes. Water temperature and pH value were recorded. The water samples were kept in an icebox and transferred to the laboratory for further analysis [14].

### 2.2. Isolation of Bacterial Strains

The water samples were subjected to tenfold serial dilution (10^0^, 10^−1^, 10^−2^, 10^−3^, 10^−4^) and spread on Reasoner's 2A (R2A) agar [15], followed by overnight incubation at 28 °C. R2A was selected in this isolation process due to the fact R2A is used to study bacteria which normally inhabit potable water [15]. Pure colonies were obtained by streaking repeatedly on Trypticase Soy Agar (TSA) and incubating at 28 °C for 24 h. Characteristics of the pure colony, for instance the shape, size, color and others visual properties were recorded.

### 2.3. Bacterial Identification Using 16S rDNA Gene Sequencing

Bacterial genomic DNA was extracted and purified using The QIAamp^®^ DNA Mini Kit (Qiagen, Germantown, MD, USA) and has been used as a template for PCR. The forward primer 27F and the reverse primer 1525R were used for PCR amplification [16]. PCR amplification parameters used were performed as previously described [17]. The comparison of gene sequences was done by using BLASTN program (GenBank databases) followed by sequence alignment and phylogenetic analyses. This analysis has been done by using Molecular Evolutionary Genetic Analysis (MEGA) Version 6 [16].

### 2.4. AHL Screening using AHL Biosensor

The presence of exogenous short chain AHLs ranging from four to eight carbons was detected by using *Chromobacterium violaceum* CV026 [18]. Screening of AHL production by bacterial isolates were performed on Luria Bertani (LB) agar. Positive control (*Erwinia carotovora* GS101) and negative control (*E. carotovora* PNP22) [19,20] were included in the detection of AHLs as controls.

### 2.5. Extraction of AHL from Spent Supernatant

Bacteria was grown overnight in LB broth buffered to pH 6.5 by using 3-(*N*-morpholino)propanesulfonic acid (MOPS, 50 mM) with shaking (220 rpm) for 18 h at 28 °C. The spent supernatant was extracted twice by using an equal volume of acidified (0.1% v/v acetate acid) ethyl acetate [21]. AHL extracts were air-dried in a fume hood before proceeding to liquid chromatography-mass spectrometry (LC/MS) analysis.

### 2.6. Identification of AHL by Triple Quadrupole Liquid Chromatography Mass Spectrometry (LC/MS)

For the analysis of LC/MS, Agilent 1290 Infinity LC system (Agilent Technologies, Santa Clara, CA, USA) equipped with an Agilent ZORBAX Rapid Resolution High Definition SB-C18 Threaded Column (2.1 mm × 50 mm, with packed particle size of 1.8 μm) was used [7]. AHL extract was resuspended in acetonitrile. The parameters for the identification of AHL by Triple Quadrupole Liquid Chromatography Mass Spectrometry (LC/MS) were previously described [7]. The presence of AHLs was confirmed by MS data analysis using the Agilent Mass Hunter software version B.05.00. Analysis was based on the retention index and the comparison of the EI mass spectra with AHL standards.

## 3. Results and Discussion

### 3.1. Bacterial Identification

**Figure 2.**
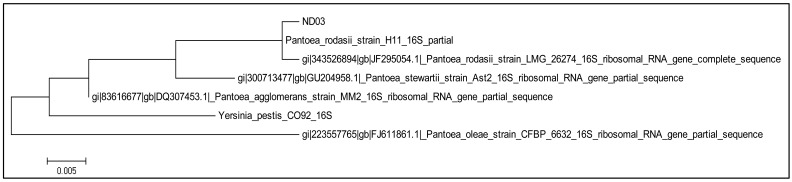
Phylogenetics analysis of strain ND03. The tree with the highest log likelihood (−917.0500) is shown. The tree is drawn to scale, with branch lengths measured in the number of substitutions per site.

Pure colonies of strain ND03 was obtained after successive streaking on growth media several times. Strain ND03 was identified analyzing its 16S rDNA nucleotides gene sequences and strain ND03 showed 99% similar to *Pantoea rodasii* strain H11. The result obtained was based on BLAST against the NCBI database and phylogenetic analysis of 16S rDNA nucleotide sequence (Figure 2). The evolutionary history was inferred by using the Maximum Likelihood method based on the Tamura-Nei model. Initial tree(s) for the heuristic search were obtained automatically by applying Neighbor-Join and BioNJ algorithms to a matrix of pairwise distances estimated using the Maximum Composite Likelihood (MCL) approach, and then selecting the topology with superior log likelihood value. There were a total of 465 positions in the final dataset. Evolutionary analyses were conducted in MEGA6. All positions containing gaps and missing data were eliminated.

### 3.2. Production of AHL by Strain ND03

*Pantoea* species members are normally plant pathogens and produces N-acylhomoserine lactones to expressly control the pathogenicity against the plant host [22]. As reported previously by Morohoshi and colleagues, the plant pathogen *Pantoea ananatis* produces *N*-hexanoyl-l-homoserine lactone (C6-HSL) and *N*-(3-oxohexanoyl)-l-homoserine lactone (3-oxo-C6 HSL) as its signaling molecules [22]. *P. ananatis* is reported as a common colonist of wheat heads during ripening and often causes “centre rot” disease in onion plants that causes crop yield reduction and postharvest losses [22]. Another member of *Pantoea, P. stewartii*, causes soft rot diseases on many plants by induction of various exoenzymes and plant tissue maceration by the production of signaling molecules such as 3-oxo-C6 HSL [23].

**Figure 3.**
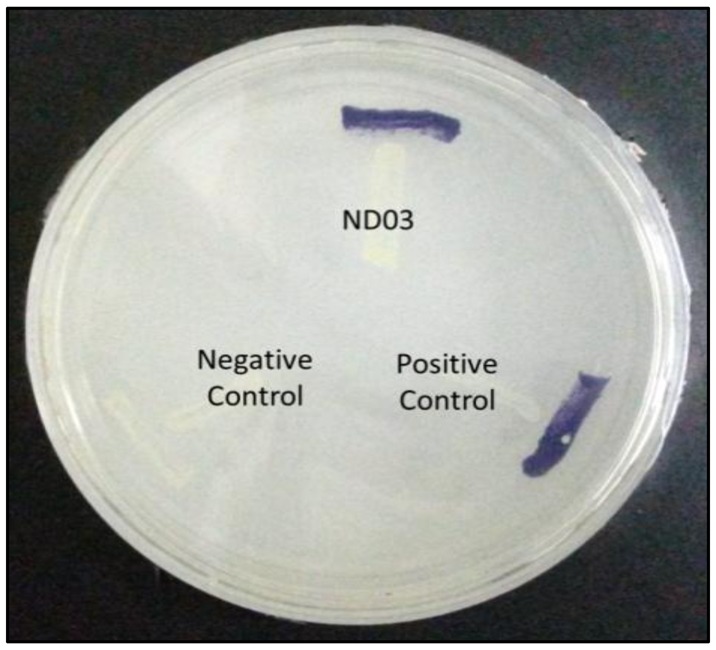
Detection of AHL production by *Pantoea rodasii* strain ND03. *E. carotovora* GS101 (Positive control) and *E. carotovora* PNP22 (Negative control) was used as positive and negative controls, respectively. The formation of purple pigmentation shows the production of short chain AHLs.

**Figure 4.**
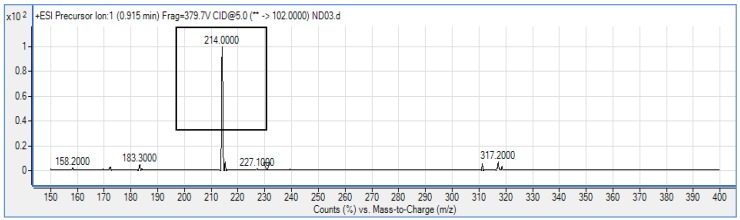
Mass spectrum of AHLs extracted from the spent supernatant of *Pantoea rodasii* strain ND03 by Triple Quadrupole LC/MS. Strain ND03 produced 3-oxo-C6-HSL (*m/z* 214.0000) (boxed).

*P. rodasii* strain ND03 was screened for the production of AHLs using CV026 whereby this isolate triggered violacein (purple pigmentation) production, suggesting our isolate produced short chain AHLs (Figure 3). In this study, CV026 was employed due to its speed and accuracy in detecting AHLs. This biosensor carries a defective AHL synthase but contain a functional LuxR-family protein (CviR) that is able to positively regulate the violacein pigment production [18]. The positive control *E. carotovora* GS101 used in this study produces 3-oxo-C6 HSL that can be detected by CV026 [20]. In order to identify the AHL produced, high resolution triple quadrupole LC/MS system was used. Using LC/MS, it is confirmed that *P. rodasii* strain ND03 produced 3-oxo-C6-HSL (*m/z* 214.0000) (Figure 4).

The detection of the AHL 3-oxo-C6-HSL in *P. rodasii* is the first report in this finding. According to research done by Brady and colleagues, *Pantoea rodasii* is a novel species which is closest related to *Pantoea dispersa, Pantoea eucrina* and *Pantoea cypripedii* based on partial sequences of *gyrB, rpoB, infB* and *atpD* using multilocus sequence analysis (MLSA) [24]. Hence, there is limited information about QS of these bacteria species. Overall, the knowledge about mechanism of QS in *Pantoea* sp. is still at its infancy. Further research in this area should be conducted in order to understand the QS circuits in this isolate.

## 4. Conclusions/Outlook

The identity of strain ND03 was confirmed as *P. rodasii* by 16S rDNA nucleotide sequencing analysis. This bacterial strain also showed positive QS activity by producing a short chain AHL, namely 3-oxo-C6-HSL which has been confirmed by LC/MS analysis. This is the first documentation of this fact nd it will pave the way to understanding better the QS circuits in this bacterium. Our future plan is to sequence the entire genome of this isolate in order to identify its luxI and luxR homologues and the QS circuit.
